# Neuroprotective effects of minocycline and KML29, a potent inhibitor of monoacylglycerol lipase, in an experimental stroke model: a small-animal positron emission tomography study

**DOI:** 10.7150/thno.64320

**Published:** 2021-09-13

**Authors:** Tomoteru Yamasaki, Akiko Hatori, Yiding Zhang, Wakana Mori, Yusuke Kurihara, Masanao Ogawa, Hidekatsu Wakizaka, Jian Rong, Lu Wang, Steven Liang, Ming-Rong Zhang

**Affiliations:** 1 Department of Advanced Nuclear Medicine Sciences, Institute of Quantum Medical Sciences, National Institutes for Quantum and Radiological Science and Technology, 4-9-1 Anagawa, Inage-ku, Chiba 263-8555, Japan.; 2 SHI Accelerator Service Co., 1-17-6 Osaki, Shinagawa-ku, Tokyo 141-0032, Japan.; 3 Division of Nuclear Medicine and Molecular Imaging, Massachusetts General Hospital, and Department of Radiology, Harvard Medical School, Boston, Massachusetts 02114, United States.; 4 Center of Cyclotron and PET Radiopharmaceuticals, Department of Nuclear Medicine and PET/CT-MRI Center, The First Affiliated Hospital of Jinan University, 613 West Huangpu Road, Tianhe District, Guangzhou 510630, China.

**Keywords:** MAGL, TSPO, PET, Minocycline, KML29

## Abstract

Hypoxia caused by ischemia induces acidosis and neuroexcitotoxicity, resulting in neuronal death in the central nervous system (CNS). Monoacylglycerol lipase (MAGL) is a modulator of 2-arachidonoylglycerol (2-AG), which is involved in retrograde inhibition of glutamate release in the endocannabinoid system. In the present study, we used positron emission tomography (PET) to monitor MAGL-positive neurons and neuroinflammation in the brains of ischemic rats. Additionally, we performed PET imaging to evaluate the neuroprotective effects of an MAGL inhibitor in an ischemic injury model.

**Methods:** Ischemic-injury rat models were induced by intraluminal right middle cerebral artery occlusion (MCAO). PET studies of the brains of the ischemic rats were performed at several experimental time points (pre-occlusion, days 2, 4, and 7 after the MCAO surgery) using [^11^C]SAR127303 for MAGL and [^18^F]FEBMP for 18 kDa translocator protein (TSPO, a hall-mark of neuroinflammation). Medication using minocycline (a well-known neuroprotective agent) or KML29 (a potent MAGL inhibitor) was given immediately after the MCAO surgery and then daily over the subsequent three days.

**Results:** PET imaging of the ischemic rats using [^11^C]SAR127303 showed an acute decline of radioactive accumulation in the ipsilateral side at two days after MCAO surgery (ratio of the area under the curve between the ipsilateral and contralateral sides: 0.49 ± 0.04 in the cortex and 0.73 ± 0.02 in the striatum). PET imaging with [^18^F]FEBMP, however, showed a moderate increase in accumulation of radioactivity in the ipsilateral hemisphere on day 2 (1.36 ± 0.11), and further increases on day 4 (1.72 ± 0.15) and day 7 (1.99 ± 0.06). Treatment with minocycline or KML29 eased the decline in radioactive accumulation of [^11^C]SAR127303 for MAGL (minocycline-treated group: 0.82 ± 0.06 in the cortex and 0.81 ± 0.05 in the striatum; KML29-treated group: 0.72 ± 0.07 in the cortex and 0.88 ± 0.04 in the striatum) and increased uptake of [^18^F]FEBMP for TSPO (minocycline-treated group: 1.52 ± 0.21 in the cortex and 1.56 ± 0.11 in the striatum; KML29-treated group: 1.63 ± 0.09 in the cortex and 1.50 ± 0.17 in the striatum). In MCAO rats, minocycline treatment showed a neuroprotective effect in the sensorimotor cortex suffering from severe hypoxic injury, whereas KML29 treatment saved neurons in the striatum, including bundles of myelinated axons.

**Conclusions:** PET imaging allowed visualization of the different neuroprotective effects of minocycline and KML29, and indicated that combination pharmacotherapy using these drugs may be an effective therapy in acute ischemia.

## Introduction

Ischemia causes severe damage to the central nervous system (CNS) and brain. In the early phase of cerebral ischemia, there is a shift from aerobic to anaerobic energy metabolism because of hypoxia, resulting in intracellular hyper-accumulation of acidic sources (lactate, H^+^, and carbonic acid) 1. Intracellular acidosis stimulates excessive glutamate release into the extracellular space, which can activate glutamate receptors. Of these, metabotropic glutamate receptor subtype 1 (mGluR1) and subtype 5 (mGluR5), which are classified as group I metabotropic-type receptors, stimulate polyphosphoinositide hydrolysis via activation of phospholipase C, which induces the production of the second messengers such as inositol 1,4,5-triphosphate and diacylglycerol (DAG). These messengers trigger intracellular Ca^2+^ release from the smooth-surfaced endoplasmic reticulum 2. The resulting Ca^2+^ overloads and then triggers secondary signal cascades, activating proteases and phospholipases and producing free radicals 3. This is the typical excitotoxicity mechanism that contributes to cerebral ischemia-induced neuronal injury.

The endocannabinoid system (eCS) is a retrograde modulating system for the homeostasis of neurotransmitters in emergency conditions such as ischemia 4, 5. The eCS incorporates the endocannabinoids (eCBs), their synthetic and degradative enzymes, eCB transporters, and cannabinoid receptors (CB1 and CB2). The main eCBs in the brain are anandamide and 2-arachidonoylglycerol (2-AG). The 2-AG level in the rat brain is 1000 times higher than the anandamide level (nmol/g vs. pmol/g), suggesting that 2-AG is the primary endogenous ligand for activation of eCS in the CNS 6, 7. The ligand 2-AG is biosynthesized from DAG, which is produced by activation of mGluR1 and mGluR5 via DAG lipase. The 2-AG crosses, in a retrograde direction from postsynaptic membranes to presynaptic membranes, where it activates presynaptic CB1 receptors, resulting in the inhibition of glutamate release via modulation of Ca^2+^ or K^+^ channels 8. Therefore, eCS is a possible pharmacological target as an important modulator of ischemic injury. However, several therapeutic studies using modulators for CB1 receptor demonstrated conflicting results (neuroprotective or neurotoxic responses) in ischemia 8. Although it is clear that eCS is modulated with an exquisite balance that depends on a number of factors, the details of the mechanism by which it affords neuroprotection remain unclear.

Monoacylglycerol lipase (MAGL) is a serine hydrolase that tightly regulates 2-AG-mediated neurotransmission in the eCS, and is identified not only in pre-synaptic terminals, but also in post-synaptic terminals and astrocytes 9, 10. Several reports demonstrated that treatment with MAGL inhibitors afforded neuroprotective effects in experimental ischemia models 11, 12. Activation of MAGL induces the production of arachidonic acid (AA), which is the primary source for synthesizing the eicosanoids related to the pro-inflammatory response, resulting in the activation of microglia, which are the resident macrophages of the brain 13. Excess MAGL activation is strongly linked with neuroinflammation based on microglial activation. Therefore, MAGL has recently been noted as a novel pharmacological target to enhance neuroprotective and anti-inflammatory effects in the brain 14, 15.

Positron emission tomography (PET) is frequently used as an imaging modality or quantification tool for basic and clinical research to elucidate drug kinetics, molecular density, and distribution *in vivo*. Furthermore, PET allows for non-invasive assessment of longitudinal changes in functions of target molecules.

Recently, we developed [^11^C]carbonyl-labeled 1,1,1,3,3,3-hexafluoropropan-2-yl 4-(((4-chlorophenyl)sulfonamido)methyl)piperidine-1-carboxylate ([^11^C]SAR127303) as a selective PET probe for MAGL 16, 17. PET with [^11^C]SAR127303 would permit monitoring of the systemic state of eCS function associated with MAGL in ischemic injury.

One of the objectives of this study was to use PET to investigate time-dependent changes in the brain uptake of [^11^C]SAR127303 after ischemic injury in an experimental stroke model. Further objectives were to simultaneously monitor neuroinflammation using a selective PET ligand (2-[5-(4-[^18^F]fluoroethoxy-2-oxo-1,3-benzoxazol-3(2*H*)-yl)-*N*-methyl-*N*-phenylacetamide]: [^18^F]FEBMP 18) for 18 kDa translocator protein (TSPO), which is a hallmark of microglial activation 19-21, and to compare the results with PET imaging for MAGL in neuroinflammation. The primary purpose of this study was to use PET with [^11^C]SAR127303 and [^18^F]FEBMP to compare therapeutic effects between minocycline, a known neuroprotective agent, and KML29, a potent inhibitor for MAGL, in an ischemic rat model.

## Materials and Methods

### Ethics Statement for Animal Experiments

Animal experiments and handling were carried out in accordance with the recommendations of the Committee for the Care and Use of Laboratory Animals of the National Institutes for Quantum and Radiological Science and Technology (QST) and the ARRIVE guidelines (http://www.nc3rs.org/ARRIVE). All animal experiments were also approved by the committee of QST.

### Radiochemistry

[^11^C]SAR127303 for MAGL imaging was prepared in-house by reaction of a piperidine precursor with an [^11^C]carbonate intermediate, which resulted from [^11^C]COCl_2_ with 1,1,1,3,3,3-hexafluoropropane-2-ol in the presence of 1,2,2,6,6-pentamethylpiperidine, as reported previously 16, 17. The radiochemical purities were more than 98% and molar activities were 52-95 GBq/μmol (n = 18) at the end of synthesis (EOS).

[^18^F]FEBMP for TSPO imaging was synthesized by reacting a desmethyl precursor with [^18^F]fluoroethyl bromide in the presence of NaOH at 120 °C for 10 minutes, according to our previous report 18. The radiochemical purities and molar activities of [^18^F]FEBMP were more than 98% and 185-370 GBq/μmol (n = 18), respectively, at EOS.

### Experimental Stroke Model

Male Sprague-Dawley (SD) rats (n = 25, 6-7 weeks old, 190-230 g) were purchased from Japan SLC (Shizuoka, Japan) and kept in a temperature-controlled environment with a 12-hour light/dark cycle. The rats were allowed free access to water and a standard diet (MB-1/Funabashi Farm, Chiba, Japan), and were used in the experiments after appropriate acclimation to the housing conditions.

Transient focal ischemia was induced by intraluminal middle cerebral artery occlusion (MCAO) for 30 minutes according to the intraluminal thread model 22. Briefly, a healthy SD rat was anesthetized with 4% (v/v) isoflurane and maintained under anesthesia with 1.8% isoflurane. The right internal carotid artery was ligated. A 2.0-monofilament nylon suture was inserted (approximately 19 mm) into the internal carotid artery up to the level of the middle cerebral artery branches, and the neck incision was closed with a silk suture. The rat was allowed to regain consciousness. The rat was again anesthetized, and the filament carefully removed to allow reperfusion at 30 minutes from the time the intraluminal occlusion started. Body temperature was monitored and maintained at optimal levels throughout the surgery.

### Small-Animal PET Imaging

Each rat was anesthetized using 1.5% (v/v) isoflurane, and a 24-gauge intravenous catheter (Terumo Medical Products, Tokyo, Japan) was inserted into the tail vein. Rats were subsequently maintained under anesthesia and secured in a custom-designed chamber placed in the center of a small-animal PET scanner (Inveon; Siemens Medical Solutions, Knoxville, TN, USA). After target position adjustment for brain scanning, dynamic emission scans (in three-dimensional list mode) were acquired for 60 minutes (1 minute × 5 frames, 2 minutes × 8 frames, and 5 minutes × 8 frames). [^11^C]SAR127303 (35.2-43.8 MBq/0.16-0.25 mL, 394-490 pmol) or [^18^F]FEBMP (17.2-20.7 MBq/0.16-0.25 mL, 75-112 pmol) were injected via a tail vein catheter. During the PET scanning, the rats' body temperatures were maintained at 37 °C using a heated (40 °C) water circulation system (T/Pump TP401; Gaymar Industries, Orchard Park, NY, USA). After the PET experiments, the rats were allowed to recover from anesthesia and were taken back to the animal breeding facility.

PET dynamic images with a 0.6-mm slice thickness were reconstructed by filtered-back projection using a Hanning's filter with a Nyquist cutoff of 0.5 cycles per pixel. Images were then summed using PMOD software (version 3.4; PMOD technology). The time-activity curve (TAC) for each radioligand was acquired from volumes of interest (VOI) that were manually mapped onto the contralateral and ipsilateral hemisphere, cortex, and striatum of the brain using a rat-brain magnetic resonance imaging (MRI) template. The radioactivity was decay-corrected to the injection time and expressed as the standardized uptake value (SUV), which was normalized to the injected radioactivity and body weight. SUV was calculated according to the following formula: SUV = (radioactivity per milliliter tissue / injected radioactivity) × gram body weight. The area under the time-activity curve (AUC) was also calculated using the SUV on the TAC from 0 to 60 minutes.

### Immunohistochemistry

After the PET assessment (the second and 7th days post-occlusion), rats were sacrificed by cervical dislocation, and their brains were quickly removed and frozen with dry ice. Frozen brain samples were cut in-house into 20-μm-thick slices using a cryotome (HM560, Carl Zeiss, Oberkochen, Germany) for immunohistochemistry and cresyl violet staining. All sections used for staining were acquired from the same anatomical region (Bregma, 1.7 to 0.7 mm). After fixation in cold 4% paraformaldehyde in PBS for 15 minutes at 4 °C, endogenous peroxidase activity was blocked in methanol containing 0.3% hydrogen peroxide for 30 minutes at room temperature. Triple staining with primary antibodies was performed overnight at 4 °C using a goat anti-MAGL antibody (1:500, Abcam, Cambridge, UK), a rabbit anti-myelin basic protein (MBP) antibody (1:200, Abcam) to assess myelin and white matter tracts (WMTs), and a mouse anti-NeuN antibody (1:200, Abcam) for neuron-specific marking. For TSPO staining, a rabbit anti-mouse TSPO antibody (NP155, 1:1000) 23 for identifying activated glial cells in the brain was used as the primary antibody. After labeling with primary antibodies, brain sections were incubated with secondary antibodies, biotin-conjugated donkey anti-goat, fluorophore-conjugated donkey anti-rabbit (Alexa Fluor 647), goat anti-mouse (Alexa Fluor 546) antibodies, and goat anti-rabbit for 1 hour, followed by tyramide signal amplification using a Fluorescein System (PerkinElmer, Waltham, MA, USA). The sections were washed three times with PBS for 5 minutes after each step and mounted with ProLong antifade mountant (Thermo Fisher Scientific, Eugene, OR, USA). Fluorescent images were captured using a fluorescence microscope (BZ-X710, Keyence, Osaka, Japan). Cresyl violet staining was performed on sections adjacent to those that were immunohistochemically stained to allow observation of the staining of neurons and glial cells.

### Medication

The MCAO rats were randomly divided into three groups (n = 6 in each group): an ischemia group without treatment, and ischemia groups with minocycline or KML29 treatment. In accord with a previous report 24, minocycline hydrochloride (Sigma-Aldrich, St. Louis, MO, USA) at 10 mg/kg dissolved in 3 mL saline was injected intravenously immediately after the surgery and then daily over the subsequent three days. Similar to the minocycline treatment, 1 mg/kg KML29 (Tocris bioscience, Minneapolis, MN, USA), a potent covalent inhibitor of MAGL, was administered intravenously for three days. PET data were acquired from the rats in each group (ischemia, minocycline, and KML29) on the fourth day after the medication finished.

### Statistical analysis

Data are expressed as the mean ± standard error. Comparisons were made using a one-way analysis of variance followed by Tukey's tests and two-way analysis of variance with the Bonferroni post-hoc test. The analysis was performed using GraphPad Prism 5 software (GraphPad Software, La Jolla, CA, USA). Differences between groups were considered significant when P < 0.05.

## Results

### Dynamic changes in brain uptake of radioactivity after MCAO surgery

**Figure 1:**
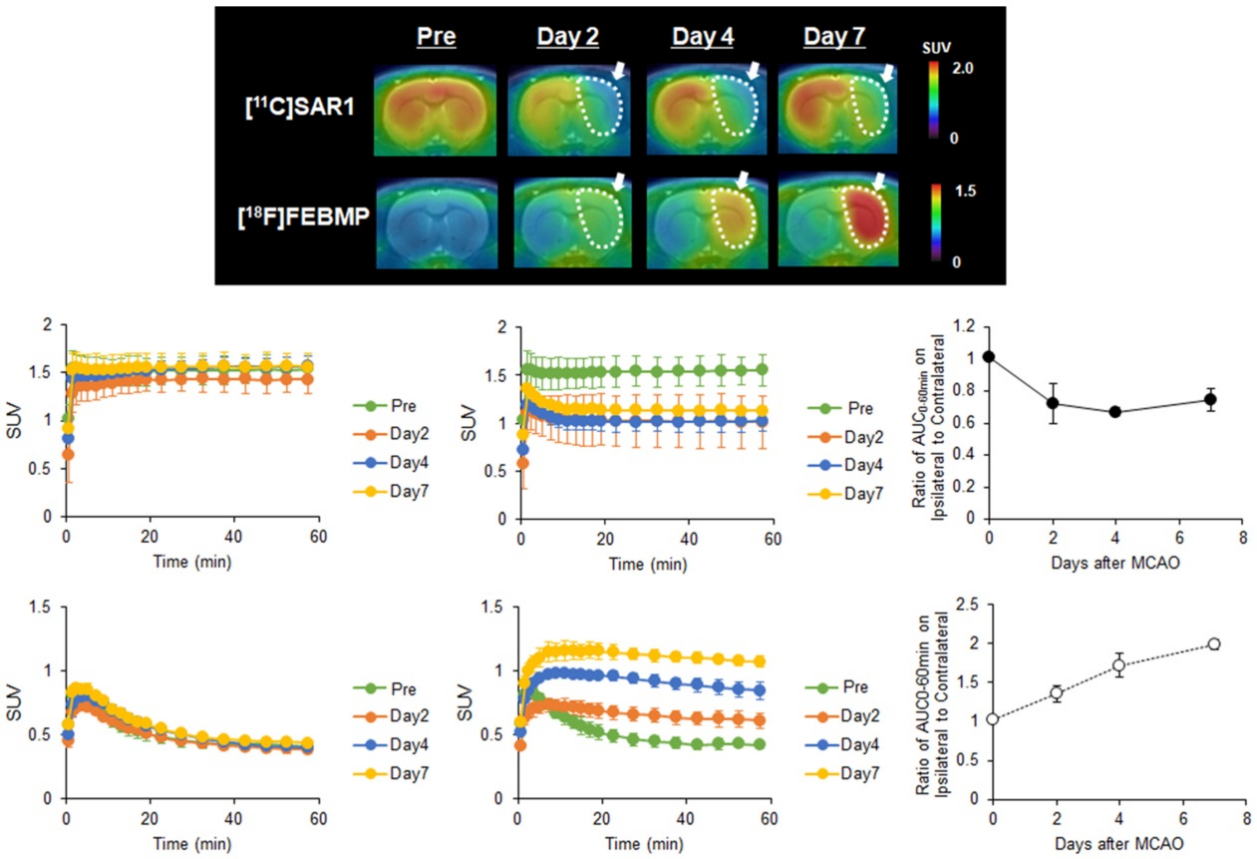
** PET imaging for MAGL and TSPO in the brain of MCAO rats. A:** Representative 0-60 minute summed PET images of [^11^C]SAR127303 (upper) and [^18^F]FEBMP (lower) in the brain pre-occlusion and days 2, 4, and 7 after MCAO surgery. The pseudo color bar represents the level of radioactive accumulation (SUV) in the brain. **B-E:** TACs of [^11^C]SAR127303 (B, C) and [^18^F]FEBMP (D, E) in the contralateral (B, D) and ipsilateral (C, E) cerebral hemisphere (n = 3 for each group). F and G: The ratio of AUC_0-60 min_ in the ipsilateral to contralateral cerebral hemisphere.

To observe time-dependent changes in MAGL activity and microglial activation after ischemic injury, we performed PET assessments with [^11^C]SAR127303 or [^18^F]FEBMP at different experimental time points (pre-occlusion, days 2, 4, and 7 after the MCAO surgery). Figure 1A shows representative PET images of [^11^C]SAR127303 and [^18^F]FEBMP in MCAO rat brains (coronal view). Compared with the PET images of [^11^C]SAR127303 in the pre-surgery animals, uptake of [^11^C]SAR127303 in the ipsilateral hemisphere was lower on days 2, 4, and 7. Conversely, increased uptake of [^18^F]FEBMP in the ipsilateral hemisphere was found starting from day 2, and this tendency in the ipsilateral hemisphere continued with time until day 7 after the MCAO surgery. Figure 1B-E shows TACs of [^11^C]SAR127303 and [^18^F]FEBMP in contralateral (B, D) and ipsilateral (C, E) areas (n = 3 for each group). Figures 1F and G show the ratios of AUC_0-60 min_ (SUV × min) between the ipsilateral and contralateral hemispheres. The ratio of [^11^C]SAR127303 showed an acute decline to 0.72 ± 0.12 at day 2 after MCAO surgery compared with the control group (1.01 ± 0.01), and then maintained a similar level at day 4 (0.67 ± 0.03) and day 7 (0.75 ± 0.07). In contrast, the ratio of [^18^F]FEBMP showed an increase to 1.36 ± 0.11 on day 2 in comparison with the control (1.02 ± 0.01), and showed further increases to 1.72 ± 0.15 at day 4 and 1.99 ± 0.06 at day 7. These time-dependent increments in the uptake of [^18^F]FEBMP after ischemic brain injury correspond with previous reports on PET-TSPO imaging, which showed microglial activation peaking around seven days after ischemia 25, 26.

### Remarkable degeneration of MAGL expression in white matter tracts

**Figure 2:**
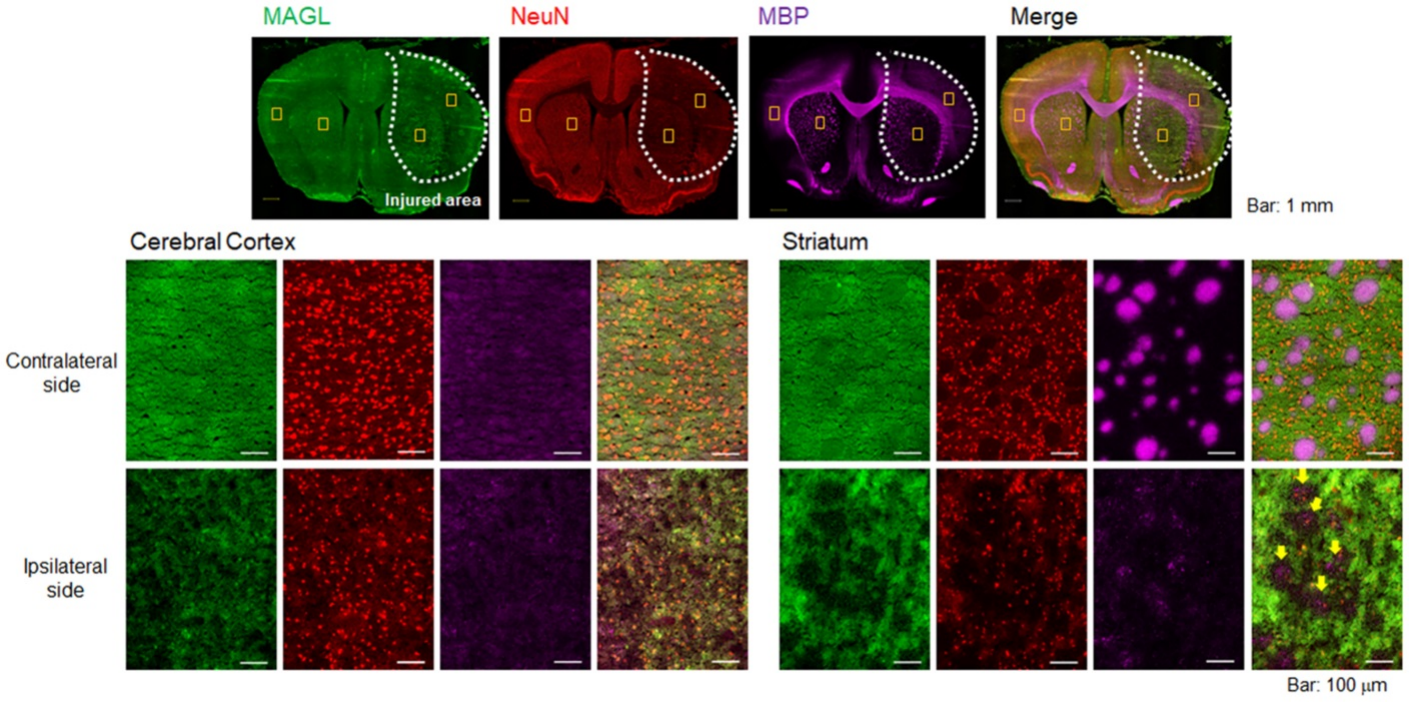
** Immunofluorescence staining of rat brain sections on day 2 after MCAO surgery.** Representative immunofluorescence images in the whole hemisphere (×4 magnification) are shown for MAGL (A; green), NeuN (B; red), and MBP (C; pink). D shows merged images of A-C. All images (**A-D**) on the top include yellow squares indicating the positions of the four close-ups. **E-H:** Close-up images (×20) for MAGL, NeuN, MBP, and merged stains in the cortex area of the contralateral hemisphere. **I-L:** Close-up images of the striatum area in the contralateral hemisphere. **M-P:** Close-up images of the cortex area in the ipsilateral hemisphere. **Q-T:** Close-up images of the striatum area in the ipsilateral hemisphere. Scale bar: 1 mm in the top images (A-D) and 50 μm in the middle and bottom images (E-T). Yellow arrows indicate a remarkable decline in MAGL signals overlapping with decreased MBP signals (T).

To evaluate pathological changes in MAGL expression, immunofluorescent staining was performed using rat brain sections obtained on day 2 after MCAO surgery. Figure 2 shows representative triple immunofluorescence images for MAGL (A; green), NeuN (B; red), and MBP (C; pink), as well as a merged image (D). The lower images (Figure 2E-T) show higher-magnification images (×20 magnification). NeuN is a well-known neuronal nuclear antigen and is a widely used marker for mature neurons, being expressed in the nucleus and cell body of most neurons but not in glial cells, oligodendrocytes, astrocytes, or microglial cells 27. The area of decreased MAGL expression overlapped considerably with the decreased area of NeuN expression (Figure 2A, B), which suggests that the MAGL-positive neurons in the ipsilateral side degenerated until at least day 2 after ischemic injury. In particular, a remarkable decline in MBP signals reflecting WMTs was observed around the central part of the injured area (Figure 2C). In the contralateral cerebral cortex (Figure 2E-H), homogenous fluorescence signals for MAGL, a high density of NeuN-stained neurons, and weak fluorescent signals for WMT were observed. In the contralateral striatum (Figure 2I-L), MAGL expression was uniformly detected, the same as in the contralateral cerebral cortex (Figure 2I). In contrast to the cerebral cortex, MBP signals indicating WMT filled with densely packed myelinated axons were clearly observed, and these did not show overlap with NeuN signals (Figure 2J and K). In the ipsilateral striatum (Figure 2Q-T), the signal for WMTs was remarkably lower, which accompanied the disappearance of fluorescence intensity indicating MAGL. However, in the ipsilateral cerebral cortex, immunoreactivity for MAGL was low, corresponding with decreased NeuN-signals. Taken together, MAGL was widely expressed not only in neurons, but also in WMTs, including bundles of myelinated axons, and therefore MAGL expression is reflective of the extent of neuronal death in the early phase of ischemic brain injury.

### Neuroprotective effect of KML29 in the early phase of ischemia

**Figure 3:**
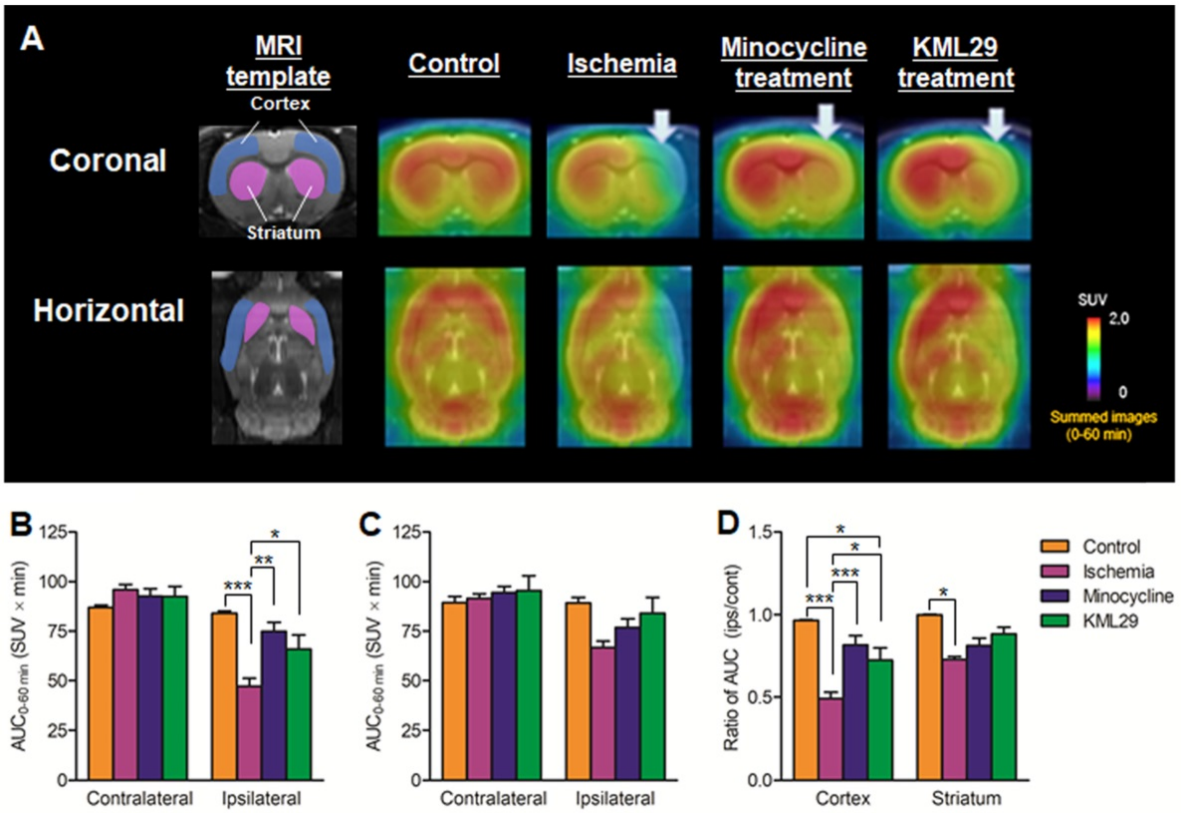
** PET imaging for MAGL in the brains of MCAO rats treated with minocycline or KML-29, or without medication.** PET images were averaged between 0 and 60 minutes after injection of [^11^C]SAR127303 and exhibited as coronal and horizontal views for healthy control, ischemia, minocycline-treated, and KML29-treated rats. The AUC_0-60 min_ values in regions of interests (cortex: **B**; striatum: **C**) were calculated from time-activity curves of the contralateral and ipsilateral sides in each rat. The ratio of AUC_0-60 min_ was calculated by dividing the AUC_0-60 min_ value of the ipsilateral side by the AUC_0-60 min_ of the contralateral side in each region of interest (**D**). Radioactivity in PET images is expressed as SUV. Values are presented are presented as the mean ± SEM. *P < 0.05, **P < 0.01, and ***P < 0.001.

To compare the neuroprotective effect of MAGL inhibitor with that of the known neuroprotective agent minocycline, medication with KML29, a commercially available inhibitor of MAGL, was performed in the rats just after the MCAO surgery. Neuronal dysfunction associated with MAGL and neuroinflammation after ischemic injury were visualized by PET assessments with [^11^C]SAR127303 and [^18^F]FEBMP on the fourth day after MCAO surgery. Figure 3 shows MRI template for VOI localization and representative PET images obtained from [^11^C]SAR127303, regional AUC_0-60min_ values (cortex and striatum), and the ratios of AUC_0-60 min_ (ipsilateral side versus contralateral side) of [^11^C]SAR127303 in the brains of control, ischemia, medication with minocycline, and medication with KML29 rats. Treatment with KML29 reduced the area showing lower radioactivity in the ipsilateral region, similar to the minocycline-treated subject. In the ipsilateral side of the ischemia rat with no medication, the AUC ratios in the cortex and striatum were 0.49 ± 0.04 and 0.73 ± 0.02, respectively. The corresponding ratios were improved to 0.82 ± 0.06 in the cortex and 0.81 ± 0.05 in the striatum by minocycline treatment and 0.72 ± 0.07 in the cortex and 0.88 ± 0.04 in the striatum by KML29 treatment, respectively. Notably, the AUC ratio in the cortex of rat treated with medication was dramatically improved with significant differences (minocycline: P < 0.001; KML29: P < 0.05), compared to that of ischemic rat.

**Figure 4:**
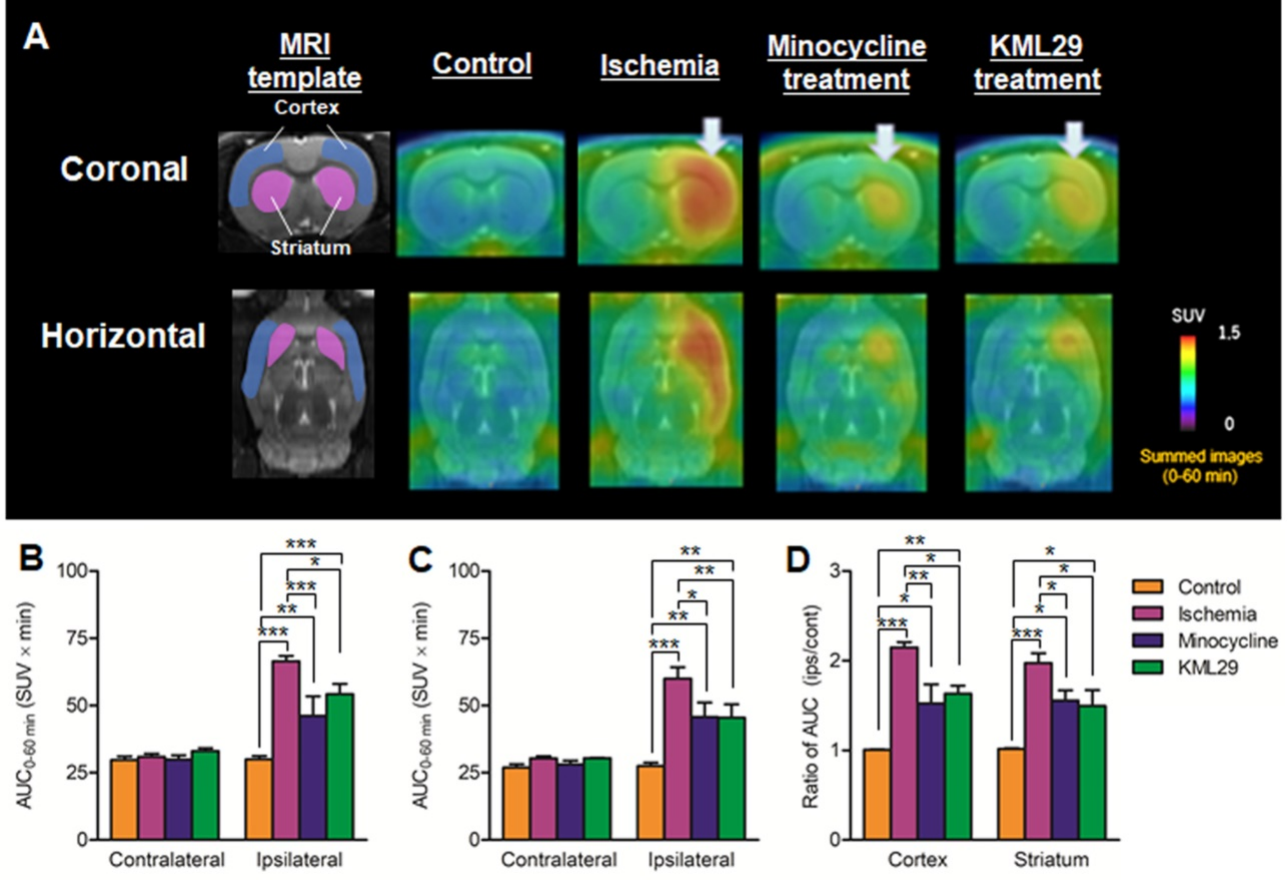
** PET imaging for TSPO in the brain of MCAO rats with or without medication treatment.** PET images were summed between 0 and 60 minutes after injection of [^18^F]FEBMP and are shown as coronal and horizontal slices for healthy control, ischemia, minocycline-treated, and KML29-treated rats. The AUC_0-60 min_ values in regions of interests (cortex: **B**; striatum: **C**) were calculated from time-activity curves of the contralateral and ipsilateral sides in each rat. The ratio of AUC_0-60 min_ was calculated by dividing the AUC_0-60 min_ value of the ipsilateral side by the AUC_0-60 min_ of the contralateral side in each region of interest (**D**). Radioactivity in PET images is expressed as SUV. Values are presented are presented as the mean ± SEM. *P < 0.05, **P < 0.01, and ***P < 0.001.

Figure 4 shows results of [^18^F]FEBMP with TSPO that are parallel to those of [^11^C]SAR127303 with MAGL. Radioactivity in the control rat was very low in the whole brain, while the radioactivity in the ischemia rat without medication showed high levels in the ipsilateral striatum and cerebral cortex. The AUC ratios in the cortex and striatum were 1.01 ± 0.01 and 1.02 ± 0.01 for control rats and 2.15 ± 0.06 and 1.97 ± 0.11 for ischemia rats. In the MCAO rats with medication, the AUC ratios in the cortex and striatum were 1.52 ± 0.21 and 1.56 ± 0.11 for minocycline-treated rats and 1.63 ± 0.09 and 1.50 ± 0.17 for KML29-treated rats. Compared with the untreated ischemic rats, the ischemic rats treated with minocycline or KML29 showed significantly lower uptake of radioactive [^18^F]FEBMP in the ipsilateral area (P < 0.05). These results indicate that treatment with minocycline or KML29 in the early phase of ischemic injury lessened the severity of neuroinflammation.

### Pathological evaluation of ischemic injury

**Figure 5:**
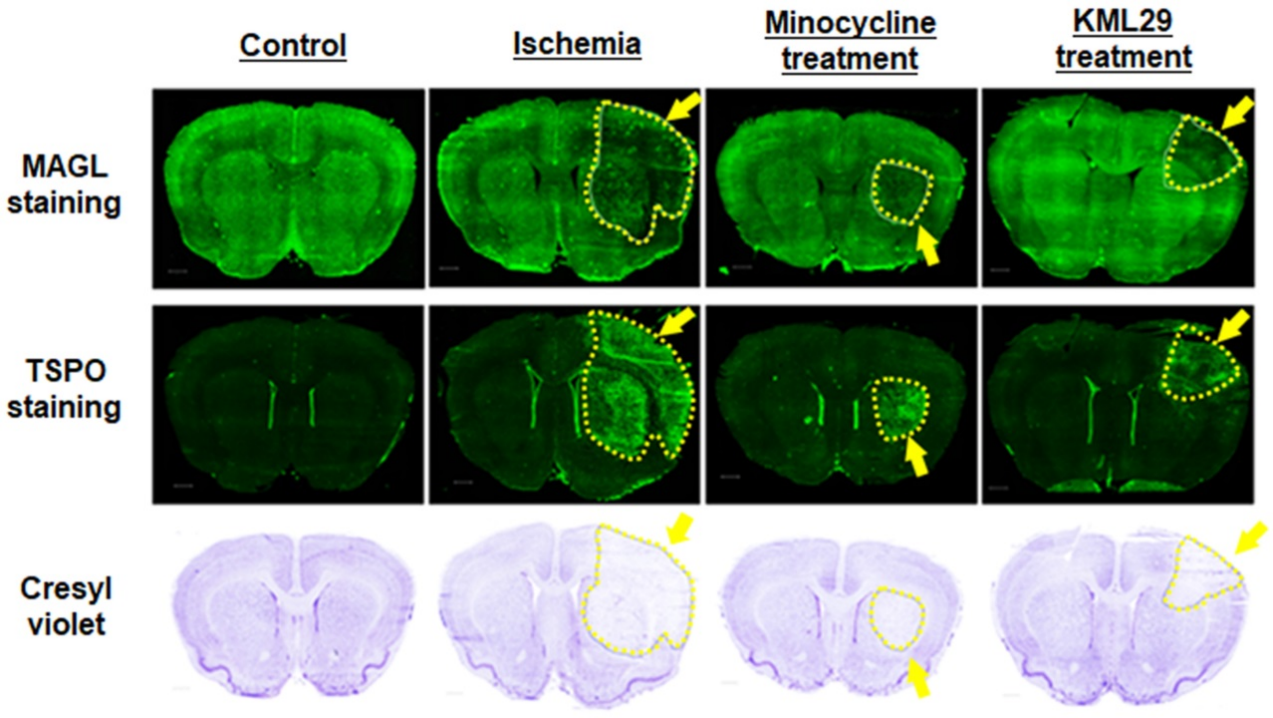
** Histological analysis of the neuroprotective effect of medication. A-D:** Immunofluorescence staining for MAGL in brain sections of control, ischemia, minocycline-treated, and KML-29-treated rats. **E-H:** Immunofluorescence staining for TSPO in brain sections of each rat. Cresyl violet **I-L:** staining for neurons in coronal brain sections of each rat. Yellow indicates neuronal injured areas. Scale bar: 1 mm.

Figure 5 shows immunofluorescence staining for MAGL (top) and TSPO (middle) and cresyl violet staining for neurons (bottom) in brain sections of rats divided into four groups (control, ischemia, minocycline-treatment, and KML29-treatment). In the brain sections of healthy control rats, strong fluorescence signals reflecting high MAGL expression were shown in the striatum and cerebral cortex, whereas fluorescence signals for TSPO expression were negligible throughout the brain section. In the brain sections of the ischemia group, the fluorescence intensity for MAGL was lower in the cerebral cortex and striatum of the ipsilateral side, whereas fluorescence signals for TSPO were enhanced in the same regions, strongly suggesting the degeneration of neurons. The area of neuronal degeneration accompanied with neuroinflammation was reduced by treatment with minocycline or KML29. The area expressing MAGL accorded well with the neuron area that was detected by cresyl violet staining, and a similar pattern of reduced neurons and decreased MAGL was seen in the ipsilateral side of ischemia brains. There was also a difference in the area of neuroprotection between minocycline and KML29 treatments. In the brain sections of the minocycline-treated rat, the neuroprotection effects were mainly detected in the cerebral cortex area, whereas in the brain sections of the KML29-treated rat, reduction of neuronal degeneration was observed in the striatal area.

## Discussion

In the present study, we successfully used PET with [^11^C]SAR127303 to monitor neuronal dysfunction associated with MAGL in the early phase of neuroinflammation accompanied by ischemic injury. Moreover, medication with an MAGL inhibitor demonstrated neuroprotection against ischemic injury in the neuronal core different from that provided by minocycline, a well-known neuroprotective agent.

First, to investigate the relationship between MAGL expression and neuroinflammation, longitudinal PET assessments using [^11^C]SAR127303 for MAGL and [^18^F]FEBMP for TSPO (a hall-mark of microglial activation) were conducted in the brains of MCAO rats. Among the possible TSPO radioligands 28, we selected [^18^F]FEBMP because it is a promising radioprobe with little influence on the binding affinity for the TPSO polymorphism 18. In the PET imaging with [^11^C]SAR127303, a remarkable decrease in uptake of radioactivity was shown in the ipsilateral area on day 2 (Figure 1). Similarly, immunohistochemistry for MAGL expression in the brain sections of day 2 MCAO rat exhibited low signal in the cortex and striatum of the ipsilateral side. At the same time, neuronal loss was also observed in the same area (Figure 2). These results reflect the decrease in uptake of [^11^C]SAR127303 in the PET images, which was caused by a remarkable decline in MAGL expression accompanied with degeneration of neurons. Several studies on cerebral ischemia-reperfusion injury showed that pro-apoptosis factors elevated to a peak at 24-48 hours of reperfusion 29-31. However, a high level of uptake of [^18^F]FEBMP was observed from days 4 to 7 in the ischemic area (Figure 1). The radioactive uptake of [^18^F]FEBMP would be likely to reach a maximum level at day 7, because the expression of TSPO is known to peak from day 7 to day 11 after ischemia 25, 32. Interestingly, a slight upward trend of [^11^C]SAR127303 was observed on day 7 when there was a maximum TSPO expression (Figure 1). It was reported that cytoplasmic outflow following neuronal cell death induced microglial activation via excessive 2-AG production from astrocyte and subsequently excessive 2-AG production was limited by astrocytic MAGL 33, 34. Therefore, a slight increase of [^11^C]SAR127303 uptake on day 7 in the present study may indicate that MAGL expression in astrocyte is upregulated to attenuate microglial activation. Taken together, the dysfunctions in neurons had occurred by two days after the MCAO surgery, when microglial activation was still gentle, and the neuroinflammation process subsequently became more aggressive from day 4 to day 7 after cerebral ischemia-reperfusion injury. More importantly, it was demonstrated that MAGL is not expressed in activated microglial cells, a finding that corresponds with previous research 9, 10. Therefore, PET with [^11^C]SAR127303 allowed normal and abnormal conditions to be distinguished in neurons in the acute phase of cerebral injury.

As uptake of [^11^C]SAR127303 on the ipsilateral side had reduced considerably at day 2 after MCAO, the pharmacotherapy for cerebral ischemic-reperfusion injury was carried out as soon as possible after reperfusion, to simulate the clinical requirement. In the present study, minocycline or KML29 administration was commenced just after the reperfusion. Minocycline, a tetracycline family antibiotic, has been reported to reduce the infarct area in a transient MCAO rat model by inhibiting microglial activation 35, 36. Multiple mechanisms for the neuroprotective effect of minocycline have been reported, including potent inhibition of microglial activation, interception of the activities of NOS, COX-2, and MMPs enzymes, and enhancement of the anti-apoptotic Bcl-2/cytochrome c pathway 36-38. However, KML29, as a potent and selective MAGL inhibitor, increased 2-AG levels and concomitantly reduced AA levels 39. The neuroprotective effects of MAGL inhibition are enhancement of the retrograde restriction of glutamate release via eCS and reduced AA production. The PET results after medication indicated that treatment with minocycline or KML29 inhibited a reduction in [^11^C]SAR127303 uptake and increased [^18^F]FEBMP uptake in the ipsilateral brain regions, which implies significant neuroprotection from both medications (Figures 3 and 4).

It should be noted that the neuroprotected areas differed between minocycline and KML29 treatments. In the minocycline-treated group, neuronal injury and neuroinflammation remained in the striatum. However, in the KML29-treated group, degeneration of MAGL expression and upregulation of TSPO expression were observed in a part of the cerebral cortex. As shown in Figure 2, although MAGL is widely expressed throughout the whole brain, the decline of MAGL expression in the ipsilateral area was remarkable in the striatal WMTs, including the bundles of myelinated axons. Most axons within the striatum WMTs arise from excitatory pyramidal neurons in the cerebral cortex and stretch toward the internal capsule 40, 41. MAGL was reported to be expressed in both corticothalamic and thalamocortical tracts, and to traverse the striatum to provide fine regulation of 2-AG availability for the developing somatosensory circuit 42. Additionally, approximately 95% of the neurons in the striatum are GABAergic inhibitory medium spiny neurons (MSNs) 40. MSNs receive excitatory glutamatergic inputs from the cerebral cortex and thalamus, and a modulatory dopaminergic innervation from the midbrain 43. The 2-AG-selective CB1 receptor is expressed at high levels in GABAergic axon terminals of MSNs and parvalbumin-positive interneurons, while it is low in excitatory corticostriatal afferents 44.

**Figure 6:**
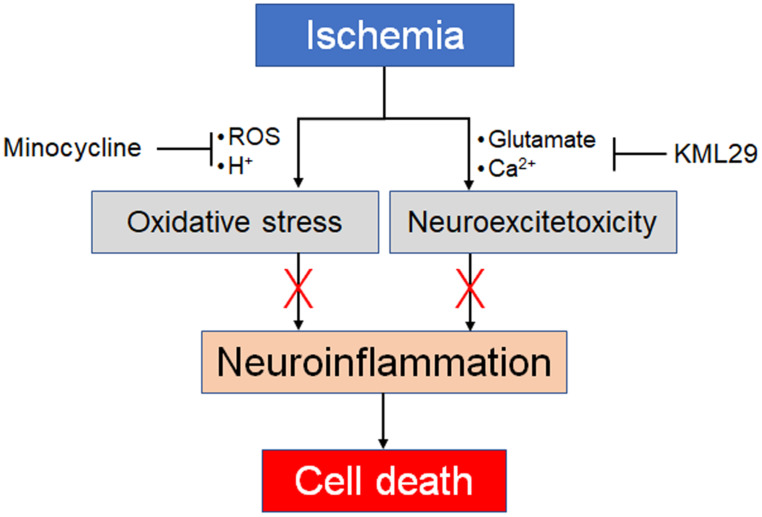
Our proposal for combination pharmacotherapy against ischemia. Ischemia induces two main factors, oxidative stress and neuroexcitotoxicity, which both lead to neuroinflammation. Therefore, neuroprotective effects should be enhanced by preventing both factors by using an anti-oxidative agent and an MAGL inhibitor.

Taken together, the present results strongly suggest different neuroprotective effects between minocycline and KML29 in the early phase of ischemic injury. Minocycline can attenuate neuroinflammation caused by oxidative stress, and therefore medication with minocycline showed a neuroprotective effect in the severely hypoxic region, notably the sensorimotor cortex. However, minocycline could not protect neuronal cells from neuroinflammation in the ipsilateral striatum, which suggests that medication with minocycline may be of little value against neuroexcitotoxic damage. In contrast, treatment with KML29 showed remarkable neuroprotection in the striatum containing rich-bundles of myelinated axons, which suggests that KML29 could save neuronal cells from the apoptosis caused by glutamate neuroexcitotoxicity via eCS activation. It has been reported that enhancement of eCS indicated neuroprotection by preventing neuronal apoptosis and improving cognition via phosphoinositide 3-kinase/AKT signaling 45. However, medication by KML29 did not produce a neuroprotective effect in the regions suffering from severe hypoxic damage, such as the sensorimotor cortex. In seriously hypoxic areas, energy metabolism shifts from aerobic to anaerobic, which produces a lower level of adenosine triphosphate. Moreover, the accumulation of acidic sources induced by ion-channel failures may lead to metabolic acidosis, resulting in necrosis and apoptosis of neurons 46, 47. Thus, our present results directly suggest that medication for ischemic injury should combine medications for oxidative stress and neuroexcitotoxicity (Figure 6).

In conclusion, we used two PET probes to demonstrate *in vivo* monitoring of neuronal dysfunction and neuroinflammation in the brains of ischemic rats. Additionally, through our experiments applying medication during the initial ischemic injury, we propose the use of a combination pharmacotherapy against oxidative stress and neuroexcitotoxicity.

## Abbreviations

2-AG: 2-arachidonoylglycerol
AA: arachidonic acid
AUC: area under the time-activity curve
CNS: central nervous system
DAG: diacylglycerol
eCB: endocannabinoid
eCS: endocannabinoid system
EOS: end of synthesis
[^18^F]FEBMP: 2-[5-(4-[^18^F]fluoroethoxy-2-oxo-1,3-benzoxazol-3(2*H*)-yl)-*N*-methyl-*N*-phenylacetamide]
KML29: 1,1,1,3,3,3-hexafluoropropan-2-yl 4-(bis(benzo[*d*][1,3]dioxol-5-yl)(hydroxy)methyl)piperidine-1-carboxylate
[^11^C]SAR127303: 1,1,1,3,3,3-hexafluoropropan-2-yl 4-(((4-chlorophenyl)sulfonamido) methyl)piperidine-1-[^11^C]carboxylate
TSPO: 18 kDa translocator protein
mGluR1: metabotropic glutamate receptor subtype 1
mGluR5: metabotropic glutamate subtype 5
MCAO: middle cerebral artery occlusion
MAGL: monoacylglycerol lipase
MBP: myelin basic protein
MRI: magnetic resonance imaging
PET: positron emission tomography
SUV: standardized uptake value
TAC: time-activity curves
VOI: volumes of interest
WMT: white matter tracts
